# Association between malnutrition, clinical parameters and health-related quality of life in elderly hospitalized patients with Parkinson’s disease: A cross-sectional study

**DOI:** 10.1371/journal.pone.0232764

**Published:** 2020-05-04

**Authors:** Maria Theresa Gruber, Otto W. Witte, Julian Grosskreutz, Tino Prell

**Affiliations:** 1 Department of Neurology, Jena University Hospital, Jena, Germany; 2 Center for Healthy Ageing, Jena University Hospital, Jena, Germany; Philadelphia VA Medical Center, UNITED STATES

## Abstract

**Objective:**

This study aimed to explore the association between malnutrition, clinical parameters, and health-related quality of life in elderly hospitalized patients with Parkinson’s disease (PD).

**Methods:**

Cross-sectional study of 92 hospitalized elderly patients with PD (mean age 73.6 ± 6.7 years) without dementia. The Mini Nutritional Assessment (MNA) was used to evaluate nutritional status. Motor impairment and non-motor symptoms burden (Movement Disorder Society-sponsored revision of the Unified Parkinson's Disease Rating Scale [MDS-UPDRS], Non-Motor Symptoms Questionnaire, and Hoehn & Yahr staging), depression (Becks Depression Inventory-II), and health-related quality of life (PD quality of life Questionnaire-39) were assessed.

**Results:**

Every second patient was malnourished or at risk of malnutrition. In the multivariable analysis, male gender, longer disease duration, higher Hoehn & Yahr and depression were associated with total MNA score. Besides non-motor symptoms and motor impairment, malnutrition was an independent predictor of poor health-related quality of life. In the multivariate analysis, malnutrition had a statistically significant effect on emotional well-being, mobility, social support, stigmatization, and cognition. The strongest association was found between malnutrition and emotional well-being.

**Conclusion:**

Elderly male persons with longer PD duration and higher disease stages are more likely to be malnourished or at risk for malnutrition. Malnutrition was mainly associated with poor emotional well-being, suggesting that treatment of depression and anxiety beside diet and physical activity can help improving nutrition status in these subjects. The MNA should not be used independent of other measures of cognition and depression in people with advanced PD.

## Background

Parkinson’s disease (PD) is a common neurodegenerative disorder characterized by motor impairment and a plethora of non-motor symptoms. People with PD are at a high risk of malnutrition [1]. In PD, malnutrition and risk of malnutrition were associated with motor impairment, disease duration, and several non-motor symptoms, such as constipation and depression [2, 3]. There are several reasons and mechanisms how clinical factors can contribute to malnutrition. Besides reduced food intake due to dysphagia, loss of smell, slow gastric emptying, side effects of drug therapy may also play a role [4]. As the disease progresses, people with PD increasingly need help with daily activities due to an increase of motor impairments (e.g. gait disturbances, falls) and the occurrence of neuropsychological problems (e.g. dementia) [5]. Therefore, in the course of the disease many patients have to be treated in hospital to optimize medical and non-medical treatment.

A gold standard for the optimal definition of malnutrition is still lacking [1]. The European Society for Clinical Nutrition and Metabolism (ESPEN) recommends, among others, the Mini Nutritional Assessment (MNA) for screening malnutrition and the risk of developing malnutrition. It includes physical and mental aspects that frequently affect the nutritional status among elderly people in home-care programs, nursing, homes and hospitals [6]. The MNA detects the risk of malnutrition when albumin levels and BMI are still normal. The score is derived from six items—reduced food intake in the preceding 3 months; weight loss during the preceding 3 months; mobility; psychological stress or acute disease in the preceding 3 months; neuropsychological problems; and body mass index. The MNA is highly predictive for adverse health outcome, social functioning, length of hospital stay and mortality [7].

Malnutrition is associated with poor health-related quality of life (QoL) and differentially influences distinct domains of health-related QoL, especially well-being and mobility domains [3, 8, 9]. However, in previous studies the investigated patients with and without malnutrition showed relevant differences in terms of clinical parameters, which also influence health-related QoL, such as age, disease duration, and non-motor symptoms burden [8–10]. After correction for these cofactors, mainly depression, more severe motor symptoms and more advanced disease stage remained significant predictors of poorer nutritional status in non-hospitalized patients with PD [8, 9, 11]. However, fewer data are available for elderly subjects without cognitive deficits as well as for hospitalized patients. Given that hospitalization is more frequently necessary in advanced disease stages, we assumed that malnutrition-related factors differ in hospitalized and non-hospitalized patients with PD. With the current study, we aimed to close this gap and to answer the following three questions:

- What is the prevalence of malnutrition in hospitalized elderly patients with PD?
- To what extent does malnutrition predict health-related QoL in elderly patients with PD?
- Which domains of health-related QoL are mainly affected by malnutrition?

## Methods

### Participants and assessments

This cross-sectional study was approved by the local ethics committee of the Jena University Hospital (4572-10/15), and all patients gave their written informed consent. Data were collected from patients with PD at the beginning of their stay (day 1–3) in the neurological ward of the Department of Neurology at the Jena University Hospital (Germany). All the patients were hospitalized because of the worsening of motor function or complications (dyskinesia, falls, and medication side effects). All received a multimodal treatment by specialized therapists and medication modifications during their stay in the hospital (German Multimodale Komplexbehandlung bei Morbus Parkinson) [12]. Inclusion criteria were as follows: 60 years or older and PD diagnosis according to the Movement Disorder Society (MDS) diagnosis criteria. Exclusion criteria were as follows: PD dementia, cerebrovascular disorders, delirium, deep brain stimulation, levodopa/carbidopa enteral infusion, apomorphine infusion, unable to complete a questionnaire, special diets (calorie restriction and low protein), and gastroenterological (surgical procedure, malabsorption, and inflammatory bowel disease) or renal disorders (nephritis and severe kidney failure) in medical history. All tests were conducted during the medication ON phase.

Several clinical variables were recorded, including the MDS-sponsored revision of the Unified Parkinson's Disease Rating Scale III (MDS-UPDRS III) to assess motor function, the revised Non-Motor Symptoms Questionnaire (NMS-Quest), Hoehn and Yahr staging (H&Y), and levodopa equivalent daily dose (LEDD). Cognition was assessed using the Montreal Cognitive Assessment (MoCa); PD dementia was defined as MoCa < 21 [13]. Beck’s Depression Inventory-II (BDI) was used to quantify depressive mood. Health-related QoL was assessed with the German version of the 39-item PD QoL questionnaire (PDQ-39), which is commonly used to assess eight domains: mobility, activities of daily living, emotional well-being, stigma, social support, cognition, communication, and bodily discomfort. The PDQ-39 Summary Index (PDQ-39SI) summarized the eight dimensions as one score. A maximum score of 100 represents the worst condition. The short form Mini Nutritional Assessment (MNA) questionnaire was applied to evaluate nutritional status (www.mna-elderly.com). It was specifically designed for the elderly and is recommended by the European Society for Clinical Nutrition and Metabolism. It consists of six questions about dietary regime in the last 3 months, weight loss, immobility, recent stress period, neuropsychological disorders (depression and dementia), and body mass index (BMI) or calf circumference (MNA scores of 12–14 indicate normal nutritional status, 8–11 at risk of malnutrition, and 0–7 malnutrition) [14, 15].

Epidemiological factors, the MDS-UPDRS III, NMS-Quest, H&Y, LEDD, MoCa and disease duration were derived from the medical records. The BDI and PDQ-39 were self-reports. The MNA was assessed by MT. After exclusion of 8 people with missing data in the BDI or PDQ-39, the data from 92 subjects were used for the following analyses.

### Statistical analysis

The SPSS statistical computer package (version 25.0; IBM Corporation, USA) was used for all statistical analyses. Values are given as mean and standard deviation or median and interquartile range (IQR). Categorical variables are presented as numbers or percentages.

Multiple linear regression analysis was subsequently performed to ascertain the independent predictors of the MNA. The clinical parameters / independent variables were derived from the literature and included: age, gender, disease duration, H&Y, MDS-UPDRS III, NMS-Quest, BDI, and LEDD [2, 8–11, 16–18]. The significance level for variables entering the linear regression model was set at 0.2 and for removing from the model at 0.4.

A second linear regression was used to study the association between PDQ-39SI (dependent variable) and MNA and variables known to influence health-related QoL in PD (age, disease duration, NMS-Quest, BDI, MDS-UPDRS III, and H&Y) [19, 20]. Before regression analyses, autocorrelation (Durbin–Watson) and multicollinearity (variance inflation factor and tolerance) were excluded. A multivariate analysis of variance (MANOVA) and MANCOVA were used to study the association between MNA total score and the eight PDQ-39 domains.

Anonymized data from this study are available as supplementary material.

## Results

### Malnutrition: Prevalence and association with clinical parameters

**Fig 1:**
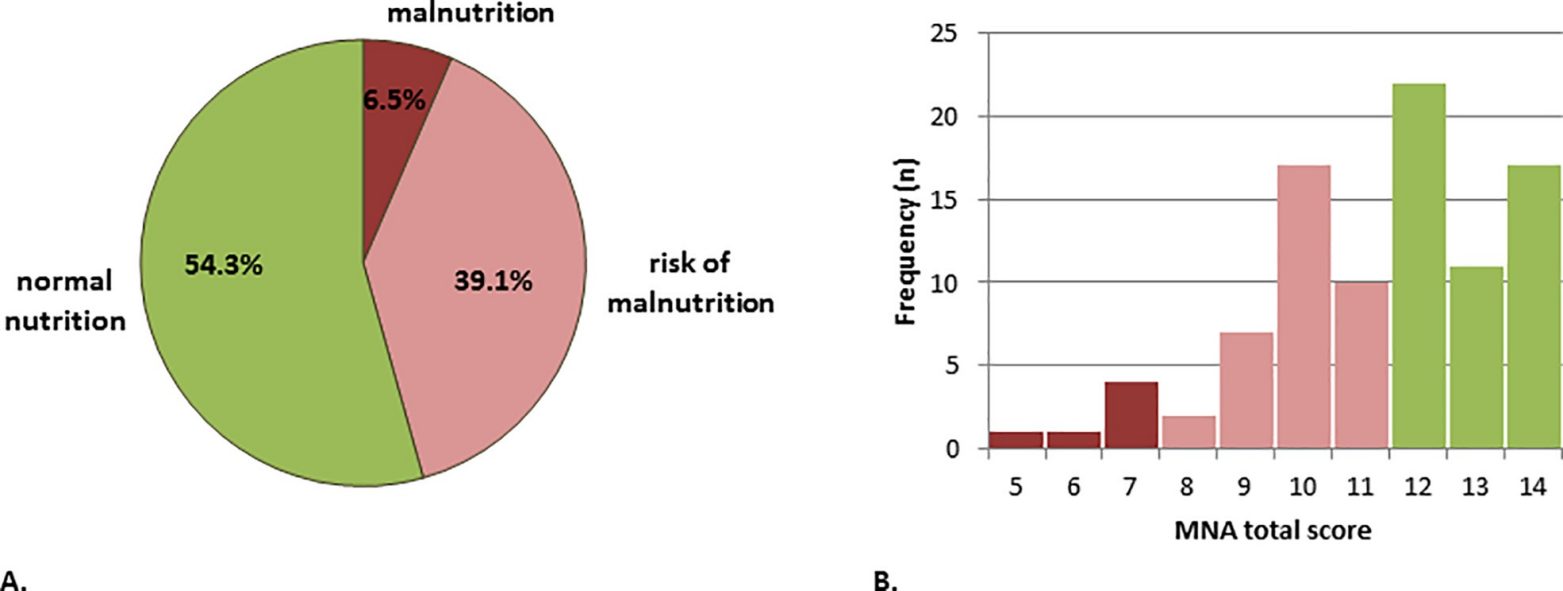
Malnutrition in patients with Parkinson’s disease according to the Mini Nutritional Assessment (MNA). (A) Prevalence of different nutritional status (*n* = 92). (B) Frequency of different MNA sum scores in the cohort.

**Fig 2:**
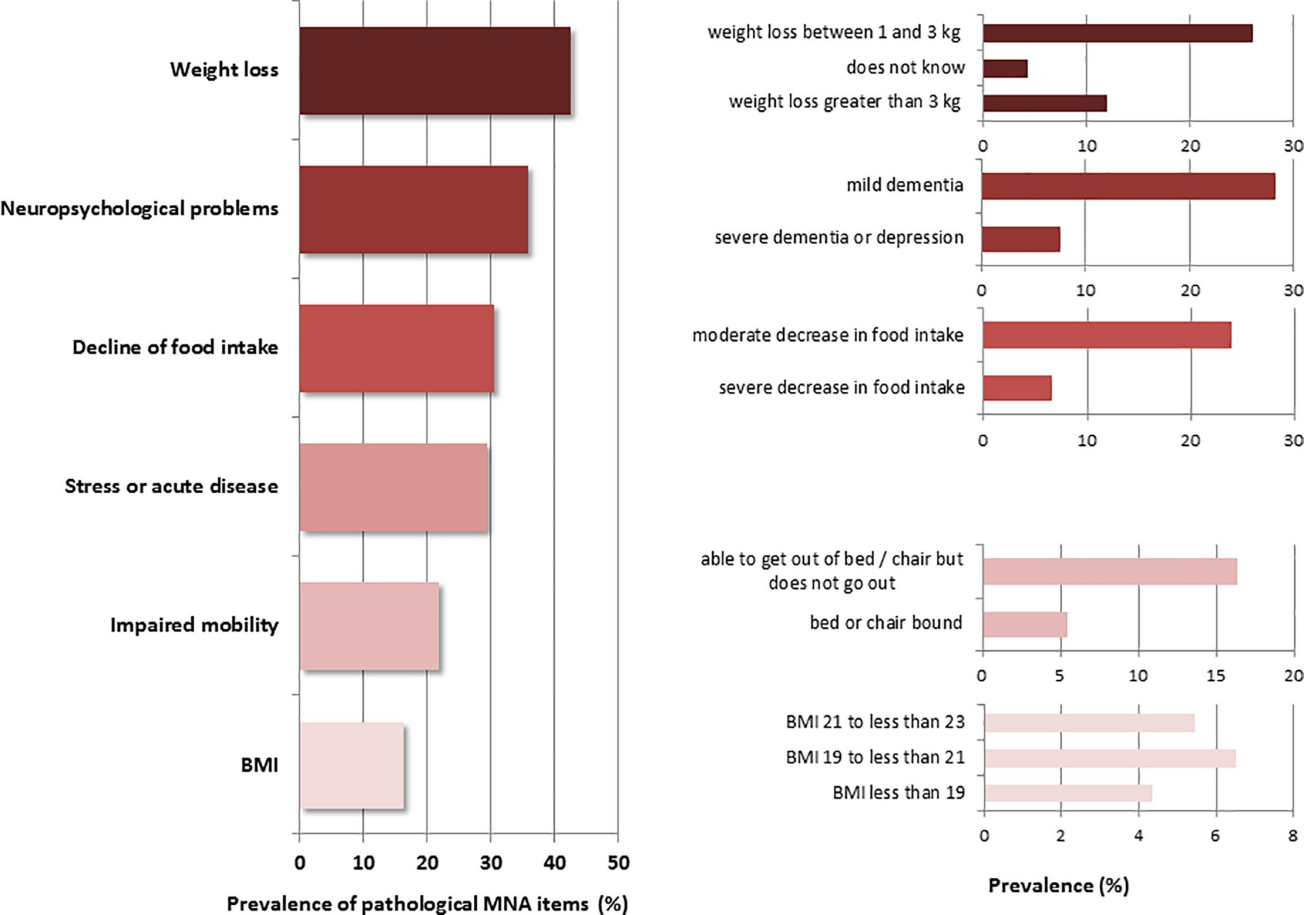
Detailed prevalence of pathological items in the Mini Nutritional Assessment (MNA). All values (%) refer to the total number of 92 subjects. BMI, body mass index.

The detailed clinical characteristics are given in Table 1. According to the MNA, every second patient was malnourished or at risk for malnutrition (Fig 1A). The histogram of the MNA sum score is shown in Fig 1B. Most patients reported weight loss (MNA item B), followed by neuropsychological problems (MNA item E) and declined food intake (MNA item A) (Fig 2).

**Table 1:** Characteristics of the cohort (n = 92).

		**Mean**	**SD**
Age		73.6	6.7
Disease duration (years)		7.9	5.7
MDS-UPDRS III (motor examination)		28.1	15.1
MDS-UPDRS IV		4.5	5.1
NMS-Quest		11.5	4.8
LEDD		670	436
Number of drugs per day		6.2	3.0
BDI total		13.7	7.6
BMI (kg/m^2^)		25.7	3.4
		**median**	**IQR**
Hoehn and Yahr stage		3.0	1.0
MNA sum score		12.0	3.0
		**n**	**%**
Gender	Female	40	43.5
	Male	52	56.5
Marital status	Married	70	76.1
	Single/divorced/widowed	22	23.9
Nutrition status	malnutrition	6	6.5
	risk of malnutrition	36	39.1
	normal nutrition	50	54.3

IQR, interquartile range; MNA, Mini Nutritional Assessment Short Form; MDS-UPDRS, Movement Disorder Society-sponsored revision of the Unified Parkinson's Disease Rating Scale; NMS-Quest, revised Non-Motor Symptoms Questionnaire; LEDD, levodopa equivalent daily dose; MoCa, Montreal Cognitive Assessment; BDI, Beck Depression Inventory; BMI, Body mass index (BMI).

In the linear regression analyses male gender, longer disease duration, higher H&Y stage, and higher BDI were found to be associated with malnutrition (Table 2).

**Table 2:** Linear regression model: Predictors of the MNA total score.

	Coefficient	Standard error	Standardised β	p	Adjusted *R*^2^
Constant	12.436	0.792			0.14
Gender (female)	-0.15	0.431	0.339	0.029	
Disease duration	-0.074	0.037	0.273	0.050	
H&Y (stage 1,2,3)	0.902	0.495	0.229	0.072	
BDI	-0.062	0.041	0.134	0.158	

### Association between malnutrition and health-related QoL

In the next step, we aimed to answer if malnutrition is associated with overall health-related QoL (PDQ-39SI) after controlling for known factors that influence PDQ-39 (age, gender, NMS-Quest, BDI, MDS-UPDRS III, and H&Y) [19, 20]. In the regression analysis, the NMS-Quest, MNA, MDS-UPDRS III, and BDI were found to be independent predictors of the PDQ-39SI (Table 3).

**Table 3:** Linear regression model: Predictors of the PDQ-39SI.

	Coefficient	Standard error	Standardised β	p	Adjusted *R*^2^
Constant	20.859	7.862			0.52
NMS-Q	1.710	0.251	0.684	<0.001	
MNA	-1.752	0.529	0.161	0.001	
MDS-UPDRS III	0.208	0.074	0.117	0.006	
BDI	0.374	0.233	0.038	0.113	

Given that malnutrition impacts the PDQ-39SI, we then analyzed the relationship between nutrition status and the PDQ-39 domains. A MANOVA was used to study the association between malnutrition and the eight PDQ-39 domains. The MANOVA revealed a significant multivariate main effect for the MNA total score on the eight PDQ-39 subdomains (*p* = 0.016; Wilk's Λ = 0.799, partial η^2^ = 0.20). However, significant univariate main effects for MNA were only found on emotional well-being (*p* < 0.001, partial η^2^ = 0.15), mobility (*p* = 0.004, partial η^2^ = 0.09), stigmatization (*p* = 0.003, partial η^2^ = 0.1), and social support (*p* = 0.043, partial η^2^ = 0.05). As indicated by the partial η^2^, the strongest association was found between malnutrition and emotional well-being. The MANCOVA was conducted to examine whether these findings could be accounted for by other medical covariates (age, disease duration, NMS-Quest, and MDS-UPDRS III). Here, disease duration (Wilks’ λ = 0.75, *p* = 0.006, partial η^2^ = 0.25), NMS-Quest (Wilks’ λ = 0.43, *p* < 0.001, partial η^2^ = 0.57), and MDS-UPDRS III (Wilks’ λ = 0.59, *p* < 0.001, partial η^2^ = 0.41), but not age (*p* = 0.13), were significant in the model. In addition, the results did not change after controlling for these variables, as there remained a significant main effect of MNA on the emotional well-being (*p* < 0.001, partial η^2^ = 0.23), mobility (*p* = 0.003, partial η^2^ = 0.11), stigmatization (*p* = 0.001, partial η^2^ = 0.13), social support (*p* = 0.02, partial η^2^ = 0.07), and in addition cognition (*p* = 0.02, partial η^2^ = 0.07).

## Discussion

Malnutrition is a highly relevant condition in elderly people with PD that favors loss of autonomy, lower quality of life, higher frequency of hospital admissions, and untimely higher mortality. The prevalence of malnutrition and risk of malnutrition we observed in hospitalized elderly patients with PD was higher than previous studies using the MNA as an outcome measure in community-dwelling elderly people [2, 8, 17, 18]. In a systematic review the prevalence of malnutrition in PD according to the MNA was between 0% and 2%, while 20% to 34% were at risk of malnutrition [18]. Most common symptom related to malnutrition was weight loss. This agrees with other studies reporting weight loss in PD [21]. Weight loss may predate diagnosis and tend to continue during PD stages [22].

In our cohort of elderly patients with PD (without dementia) malnutrition was associated with male gender, longer disease duration, higher H&Y and depressive mood. Three recent studies also investigated the association between MNA and clinical parameters. Fereshtehnejad et al. investigated 150 patients with PD (men age 60.8 ± 10.8) and found in their multivariate linear model that depression, UPDRS total score, LEDD and patients’ sex were significantly associated with the total MNA score [8]. Ongun studied 112 patients with PD (mean age = 63.7 ± 6.4) and in the multiple logistic regression analysis depression, UPDRS total score, and male gender were independently related to malnutrition [9]. The cohort studied by Tomic et al. (n = 107, mean age 70.2 ±8.6) is comparable to our cohort in terms of age, however, here only group comparisons (abnormal vs. normal nutrition) or univariate correlations between MNA and clinical parameters (age, H&Y scale, UPDRS part III, ‘off’ periods and depression) were reported and no multivariable analyses were performed [3]. In contrast to the studies of Fereshtehnejad et al. and Ongun, we did not observe an association between LEDD, NMS-Quest and malnutrition. However, from a methodological point these studies differed from our study when different measures were used (UPDRS instead of MDS-UPDRS) and the patients were younger. In summary, our and these studies show that relevant motor impairments/higher disease stages and depression are the main factors for malnutrition in PD.

We found that malnutrition is an independent predictor of the PDQ-39SI in elderly subjects with PD after controlling for other known predictors of health-related QoL, namely NMS which have large impact on QoL in elderly patients [10, 23–25]. The impact of malnutrition on health-related QoL is in line with previous studies in younger cohorts [8, 9]. In particular, emotional well-being, mobility, social support, and stigmatization were negatively influenced by malnutrition in our study. Health-related QoL was in addition studied by Fereshtehnejad et al. and Ongun using the PDQ-39. Ongun and Fereshtehnejad et al. performed univariate comparisons of the PDQ-39 domains between patients with normal and abnormal nutritional status and found higher PDQ-39 scores in all [9] or most [8] PDQ-39 domains. In contrast to other studies, we observed the strongest association between emotional well-being and malnutrition and not between mobility and malnutrition [8, 9, 26]. One has to take into account that in the studies of Ongun and Fereshtehnejad et al., significant differences of the PDQ-39 domains were observed between the poor-nutrition and normal-nutrition groups [8, 9]. However, in these two studies, both groups also significantly differed in terms of disease duration and motor and non-motor impairment, which also influence QoL. In particular, longer disease duration and higher non-motor burden might contribute to poorer health-related QoL in malnourished patients in these studies. This and the higher age in our cohort might explain these different results. The emotional well-being domain is related to symptoms of depression and long-standing anxiety [27]. Patients with depression are more likely to exhibit loss of appetite and decreased food intake, which can favor malnutrition [16, 21]. As demonstrated in our and former studies, depression and malnutrition seem to be associated in PD [11, 28, 29].

The MNA is widely used and is available in multiple languages and many studies in various settings used this tool. A large body of evidence underlines the usefulness of the MNA as screening tool in elderly people. However, despite these benefits of MNA, the sensitivity of the MNA is still debated because it has been related to a high risk of overdiagnosis of malnutrition [30–32]. Moreover, in our opinion a relevant shortcoming of the MNA in PD is lumping dementia and depression together under the term ´neuropsychological problems´. Given the high prevalence of PD dementia and depression in PD this might cause relevant bias. Moreover, the ´neuropsychological problems´ are not well operationalized in detail. In two studies among older hospitalized patients, the ´neuropsychological problems' have operationalized based on the results of Mini Mental Status Examination and the Geriatric depression score. However, both studies found different cut-offs to categorize patients into the different categories of the MNA item ´neuropsychological problems´ (no problems; mild dementia; severe depression or dementia) [33, 34]. We also observed disagreement when some patients were classified as having ´mild dementia´ in the MNA but the MoCa did not indicate PD dementia. In our study the MNA and MoCa were assessed by independent persons. The assessment of the MNA item was based on the examiner's assessment at the time the MNA was collected on the patient and not on the basis of other medical information in the medical record. Therefore, persons with subjective memory complaints can be categorized as ´mild dementia´ in the MNA although the MoCa indicates no relevant objective cognitive deficits. There are also mismatches for depression. For example, one person was scores as having ´depression´ in the MNA, but the BDI was normal. This may be due to the fact that we only recorded depression using a self-report and not using the gold standard, the interview based on DSM or ICD-10 criteria. Therefore, the MNA has limitations in some patients with PD when it is assessed independent of other measures (i.e. depression, cognition). Given the limitations when the MNA was assessed by medical staff, we assume that a MNA that is patient completed has only limited value to screen for malnutrition in advanced PD. However, our data underline that also the MNA that was completed by the nursing staff needs additional geriatric comprehensive assessment to be valid.

Our study has some more limitations. The analysis of health-related QoL and malnutrition was restricted to hospitalized patients without dementia, limiting the generalizability of our results. Moreover,—as in the other studies [8–10]—we did not perform detailed assessment of dysphagia which might have also a relevant influence on malnutrition. We restricted the analysis to the screening part of the MNA, and we cannot fully rule out that the results would differ if the full MNA would have been used. On the other hand, the sensitivity and specificity of the short form MNA are almost identical to the original MNA, confirming that the short form is valid and compares well against the full MNA [14, 15].

## Conclusion

The predictors of malnutrition and the impact on health-related QoL in our cohort of elderly hospitalized patients differ from the existing studies in younger patients. We found that elderly male persons with longer disease duration and higher disease stages are more likely to be malnourished or at risk for malnutrition. Malnutrition was mainly associated with poor emotional well-being. Further studies in elderly patients with PD should therefore answer if the treatment of depression and anxiety beside diet and physical activity can help to improve nutrition status in these subjects. The MNA should not be used independent of other measures of cognition and depression in people with advanced PD.

## Supporting information

S1 Data (SAV)
